# Data characterizing diurnal rhythms in the number of peripheral CD8α^−^ and CD8α^+^ γδ T cells in domestic pigs

**DOI:** 10.1016/j.dib.2017.12.013

**Published:** 2017-12-10

**Authors:** Larissa C. Engert, Ulrike Weiler, Volker Stefanski, Sonja S. Schmucker

**Affiliations:** Behavioral Physiology of Livestock, Institute of Animal Science, University of Hohenheim, Garbenstr. 17, 70599 Stuttgart, Germany

**Keywords:** Diurnal rhythm, Immune system, Gamma-delta T cell, CD8 alpha, *Sus scrofa domestica*

## Abstract

This data article is related to the original research article “Diurnal rhythms in peripheral blood immune cell numbers of domestic pigs” of Engert et al. [1] and describes diurnal rhythms in the number of CD8α^−^ and CD8α^+^ γδ T cells in peripheral blood of domestic pigs. Blood samples were taken from 18 animals over periods of up to 50 h and immune cell subtypes were determined by flow cytometry. Diurnal rhythmicity of cell numbers of γδ T cell subtypes was analyzed with cosinor analysis and different properties of rhythmicity (mesor, amplitude, and peak time) were calculated. In addition, associations between cell numbers of the investigated cell types in porcine blood with plasma cortisol concentration, hematocrit, and experimental conditions were identified with linear mixed model analysis.

**Specifications Table**

**Table:** 

Subject area	Biology and Agricultural Science
More specific subject area	Porcine Immunology and Chronobiology
Type of data	Figures and tables
How data was acquired	Flow cytometry (BD FACSCalibur, BD Biosciences), cosinor analysis (R version 3.1.2, R Foundation for Statistical Computing, Vienna, Austria), and linear mixed model analysis (IBM SPSS Statistics 22, IBM Deutschland, Ehningen, Germany)
Data format	Analyzed
Experimental factors	A total of 18 castrated male pigs (Piétrain × German landrace, 6-month-old) were held under a 12:12 light-dark cycle with *ad libitum* access to hay and water and concentrate feeding twice daily. Blood samples were taken every 2 h over periods of up to 50 h via indwelling vein catheters.
Experimental features	Heparinized whole blood samples were used to characterize diurnal rhythms in CD8α^−^ and CD8α^+^ γδ T cells of domestic pigs. The γδ T cell subtypes were characterized with fluorescent antibody staining and subsequent flow cytometric analysis.
Data source location	Experimental unit of the department Behavioral Physiology of Livestock, Institute of Animal Science, University of Hohenheim, 70599 Stuttgart, Germany
Data accessibility	Data are presented within this article and related to an original research article [1].

**Value of the data**

• The present data describe diurnal rhythms in the number of CD8α^−^ and CD8α^+^ γδ T cells in porcine blood and thus enhance knowledge about these specific porcine immune cell subtypes.
• The various properties of diurnal rhythmicity (mesor, amplitude, and peak time) in cell numbers of γδ T cell subtypes described here can be compared to data from other species as well as to other immune cell subtypes in domestic pigs.
• The association of CD8α^−^ and CD8α^+^ γδ T cell number in porcine blood with plasma cortisol concentration could contribute to future research about the effect of cortisol on circulating porcine γδ T cell numbers and its underlying mechanisms.

## 1 Data

**Fig. 1:**
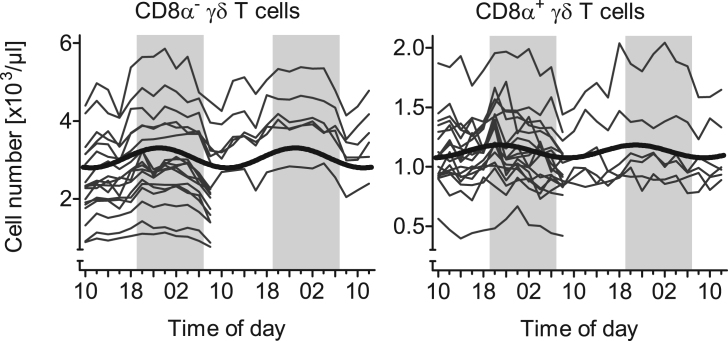
Diurnal rhythms of the cell numbers of CD8α^−^ and CD8α^+^ γδ T cells in porcine blood. Shaded areas indicate lights off. Gray lines indicate measured values of each single animal in the study (*n*=18), black curves correspond to the results of overall cosinor analyses with combined datasets of all 18 animals (significant diurnal rhythmicity at *P*<.05, refer to Table 1).

**Fig. 2:**
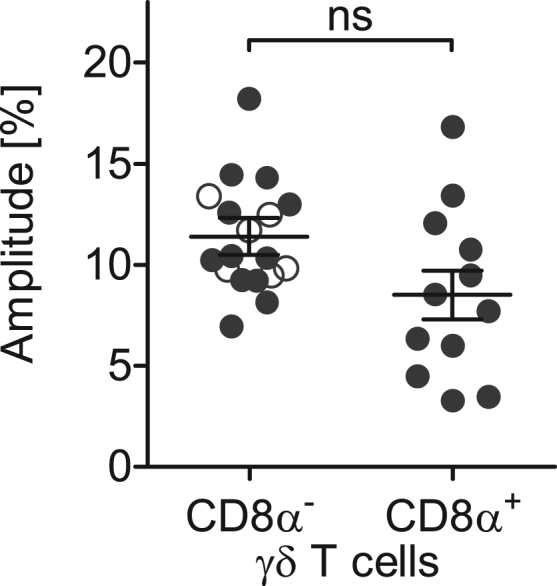
Relative amplitudes of the cell numbers of CD8α^−^ and CD8α^+^ γδ T cells in porcine blood. The statistical analysis only includes values of animals with significant (*P*<.05) diurnal rhythm in individual single cosinor analyses in both depicted γδ T cell subtypes (*n*=12 as mean±SEM, the individual values included into comparison are shown as dots, refer to Table 2; the additional data values of complete datasets, which were not included into comparison, are shown as circles); ns *P*≥.05.

**Fig. 3:**
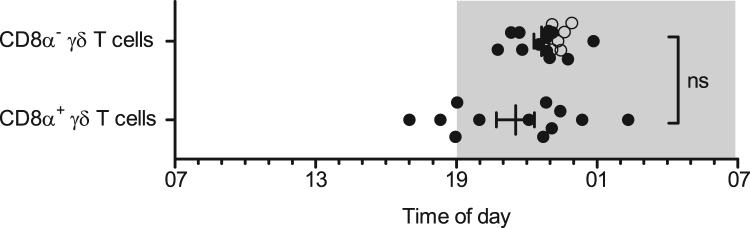
Peak times of the cell numbers of CD8α^−^ and CD8α^+^ γδ T cells in porcine blood. The statistical analysis only includes values of animals with significant (*P*<.05) diurnal rhythm in individual single cosinor analyses in both depicted γδ T cell subtypes (*n*=12 as mean±SEM, the individual values included into comparison are shown as dots, refer to Table 2; the additional data values of complete datasets, which were not included into comparison, are shown as circles); ns *P*≥.05.

In pigs and other livestock species γδ T cells are a major subset of up to 30% among all lymphocytes in blood with approximately one-third expressing CD8α [2]. The function of porcine CD8α^−^ and CD8α^+^ γδ T cells is not fully elucidated yet but studies imply potential functional differences between the two phenotypes [3], [4], [5]. The present data characterize diurnal rhythms in the cell numbers of these two subtypes of peripheral γδ T cells in domestic pigs (Fig. 1). A description of the different properties of rhythmicity (mesor, amplitude, relative amplitude, and peak time) is provided for overall cosinor analyses with combined datasets of all animals (Table 1) as well as for individual single cosinor analyses performed per animal (Table 2). Both subtypes of γδ T cells exhibited diurnal rhythms in blood cell counts with mean peak times during the dark phase. Relative amplitudes did not differ (Fig. 2; *t*(11)=2.01, *P*=0.070) between CD8α^−^ γδ T cells (95% confidence interval (CI) 9.4–13.4%) and CD8α^+^ γδ T cells (CI 5.9–11.2%). Peak times also did not differ (Fig. 3; *t*(11)=1.50, *P*=0.162) between CD8α^−^ γδ T cells (CI 21:55 h to 23:19 h) and CD8α^+^ γδ T cells (CI 19:44 h to 23:18 h).

**Table 1:** Results of overall cosinor analyses for CD8α^−^ and CD8α^+^ γδ T cells with combined datasets of all animals.

Variable	*P*^a^	*n*^b^	Mesor	Amplitude	Amplitude^c^ [%]	Peak time^d^
CD8α^−^ γδ T cells [/µl]	0.004	18	3054.8±63.6	255.3±88.4	8.4±2.9	23:00±01:22
CD8α^+^ γδ T cells [/µl]	0.032	18	1128.9±17.8	53.8±25.0	4.8±2.2	20:55±01:48

Values are presented as mean±SEM.

a Significant diurnal rhythmicity at *P*<.05.

b Number of animals in analyzed combined datasets.

c Relative amplitude (amplitude/mesor).

d Time of day±hh:mm.

**Table 2:** Results of individual single cosinor analyses for CD8α^−^ and CD8α^+^ γδ T cells per animal.

Variable	*n*^a^	Mesor	Amplitude	Amplitude^b^ [%]	Peak time^c^
CD8α^−^ γδ T cells [/µl]	18	2752.7±250.3	297.9±25.4	11.3±0.6	22:53±00:14
CD8α^+^ γδ T cells [/µl]	12	1141.0±96.1	95.3±15.9	8.5±1.2	21:31±00:49

Values are presented as mean±SEM.

a Number of animals out of 18 with significant (*P*<.05) diurnal rhythm in individual single cosinor analyses.

b Relative amplitude (amplitude/mesor).

c Time of day±hh:mm.

Linear mixed model analyses (Table 3) demonstrated that the cell numbers of CD8α^−^ and CD8α^+^ γδ T cells were positively associated with the factor light off and hematocrit but negatively associated with the factor concentrate feeding and plasma cortisol concentration. No association of the cell numbers of either γδ T cells subtypes was found with preceding sampling.

**Table 3:** Results of linear mixed model analyses.

Variable	Fixed effect	Estimate±SE	*df*^a^	*F*	*P*	Dir.^b^
CD8α^−^ γδ T cells	Intercept	1689.13±554.85	1,48.18	13.25	6.65×10^−04^	
	Light (off)	359.32±32.18	1,182.09	124.65	2.21×10^−22^	↑
	Feeding (yes)	-233.96±37.69	1,228.14	38.54	2.50×10^−09^	↓
	Cortisol (per 1 ng/ml)	-3.88±1.24	1,190.50	9.78	0.002	↓
	Hematocrit (per 1%)	25.57±10.38	1,250.84	6.07	0.014	↑
	Sampling (per sample)	-3.33±3.07	1,48.16	1.17	0.284	↔
CD8α^+^ γδ T cells^c^	Intercept	6.5272±0.2184	1,56.23	943.00	7.99×10^−37^	
	Light (off)	0.0574±0.0127	1,190.18	20.36	1.12×10^−05^	↑
	Feeding (yes)	-0.0538±0.0157	1,248.30	11.65	7.50×10^−04^	↓
	Cortisol (per 1 ng/ml)	-0.0016±0.0005	1,186.34	10.64	0.001	↓
	Hematocrit (per 1%)	0.0125±0.0043	1,269.78	8.42	0.004	↑
	Sampling (per sample)	-0.0021±0.0011	1,75.66	3.49	0.066	↔

a Numerator degrees of freedom, denominator degrees of freedom.

b Direction of estimated association: ↑ positive, ↓ negative, ↔ none.

c Logarithmic transformation of data.

## 2 Experimental design and methods

A detailed description of experimental design and methods used is provided in the accompanying research article [1]. Essential methodical information related to the present data is provided in the following sections.

### 2.1 Animals and surgery

All procedures were conducted in accordance with the German Animal Welfare Act and approved by the local Animal Welfare Ethics Committee (Regional Council Stuttgart, approval number V309/13TH). Eighteen castrated male pigs (Piétrain × German landrace, 6-month-old, weight range 92–106 kg) were included in the study and housed in a lightproof building (ambient temperature 21±1°C). Animals were kept individually but had sight and tactile contact to neighboring animals. They had *ad libitum* access to hay and water and were fed concentrate (1.2 kg/meal, ME 12 MJ/kg) twice daily at 07:30 h and 15:30 h. All animals were maintained under a 12:12 light-dark cycle (lights on 07:00 h to 19:00 h). The average illuminance at pigs’ eye level was 190 lx during the light phase (fluorescent tubes, 4000 K) and 0 lx during the dark phase. The animals were accustomed to the lighting and feeding regime for at least 8 weeks prior to the experiments and well habituated to human handling. The pigs were surgically catheterized with indwelling vein catheters (*vena cava cranialis*) at least 2 weeks prior to sampling as previously described [1].

### 2.2 Experimental protocol and sample processing

The study was subdivided into 3 different experimental trials (*n*=6 each, refer to Fig. 1 in the accompanying research article [1]). Blood sampling started at 10:00 h and was repeated every 2 h in all trials. In the first 2 trials a total of 12 blood samples were taken until 08:00 h the following day (duration 22 h each). The 3rd trial included a total of 26 blood samples and sampling ended at 12:00 h on the second following day (duration 50 h). Blood sampling at night was performed under dim light, which was switched on and off for sampling (7 lx at pigs’ eye level, 2700 K). Blood samples were immediately processed after each single sampling.

### 2.3 Flow cytometry

Heparinized whole blood (lithium heparin tubes, Sarstedt, Nümbrecht, Germany) was used to characterize immune cell subtypes by a three-color flow cytometric analysis as previously described [1]. Aliquots of 20 µl whole blood were incubated for 15 min at room temperature (RT) with different combinations of monoclonal antibodies (all obtained from SouthernBiotech, Birmingham, AL, USA). The characterization of CD8α^−^ and CD8α^+^ γδ T cells required SPRD-conjugated mouse anti-pig CD3ε antibody (clone PPT3, IgG1, 0.1 mg/ml, working dilution (WD) 1:140), FITC-conjugated mouse anti-pig CD4 antibody (clone 74-12-4, IgG2b, 0.5 mg/ml, WD 1:350), and PE-conjugated mouse anti-pig CD8α antibody (clone 76-2-11, IgG2a, 0.1 mg/ml, WD 1:350). Subsequently, the cells were incubated with BD FACS Lysing Solution (BD Biosciences, Heidelberg, Germany) for 10 min at RT, followed by two washing steps. The stained samples were maintained at 4°C until flow cytometric determination (BD FACSCalibur, BD Biosciences). At least 10,000 cells were analyzed per sample.

Flow cytometric data were processed using the software BD CellQuest Pro 6 (BD Biosciences). Initially, peripheral blood mononuclear cells (PBMC) and granulocytes were differentiated based on their forward and side scatter characteristics. According to previous research [3], [6], T cells (CD3^+^) were identified within PBMC by surface marker expression. Subsequently, CD8α^−^ γδ T cells (CD3^+^ CD4^−^ CD8α^−^) and CD8α^+^ γδ T cells (CD3^+^ CD4^−^ CD8α^dim^) were identified within T cells. The complete gating strategy within the present study is depicted in Supplementary Fig. S1 in the accompanying research article [1]. Absolute cell numbers were calculated by combining cell frequencies with total leukocyte counts, which were obtained by an automated hematology analyzer (MEK-6108G, Nihon Kohden, Rosbach, Germany) measuring whole blood samples (K3 EDTA tubes Sarstedt, Nümbrecht, Germany).

### 2.4 Statistical analyses

Diurnal rhythms were assessed using R version 3.1.2 (R Foundation for Statistical Computing, Vienna, Austria). Cosinor analysis [7] was carried out with the package *cosinor* [8]. As we were interested in diurnal rhythmicity according to the established 12:12 lighting regime, the period length was set to 24 h in all cosinor models. At first cosinor analyses were run with combined datasets of all animals to obtain overall diurnal rhythmicity in the cell numbers of CD8α^−^ and CD8α^+^ γδ T cells in porcine blood. Then, cosinor analyses were rerun for every single animal to obtain individual single diurnal rhythmicity. Diurnal rhythmicity was characterized by mesor (average value of the fitted cosine function), amplitude (half the difference between maximum and minimum of the fitted cosine function), and peak time (time of the maximum of the fitted cosine function) and was considered significant if cosinor models revealed *P*<.05 for the amplitude. The peak times were calculated by the formula −*Φ*24/(2π) using the phase shift *Φ* denoted by R and by setting 00:00 h (24 h) as reference time.

Pairwise statistical comparisons were performed with IBM SPSS Statistics 22 (IBM Deutschland, Ehningen, Germany) using paired Student's *t*-tests (two-tailed). The normality of differences was confirmed by Shapiro-Wilk tests and quantile-quantile plots.

Associations of the cell numbers of CD8α^−^ and CD8α^+^ γδ T cells in porcine blood with the potential influencing variables light, concentrate feeding, plasma cortisol concentration, hematocrit, and repeated sampling were assessed with linear mixed models (IBM SPSS Statistics 22). A detailed methodical description of linear mixed model analysis and the data of the investigated explanatory variables can be found in the accompanying research article [1]. Homoscedasticity and normality were confirmed by plotting residuals *versus* predicted values and by quantile-quantile plots of residuals, respectively. If necessary, logarithmic transformation was applied. In all linear mixed models *P*<.05 was considered significant. After backward model selection to identify relevant random effects, the following model was applied:

$\begin{array}{ccc} \begin{array}{c} y_{ij} \end{array} & = & \mu +light_{j}+concentrate\;feeding_{j}+plasma\;cortisol\;concentration_{ij}\\ & & +\;hematocrit_{ij}+sampling_{j}+animal\;identity_{i}+experimental\;trial_{i}\\ & & \begin{array}{c} +\;litter_{i}+\varepsilon_{ij} \end{array} \end{array}$

Thereby, the dependent variable *y*_*ij*_ (cell number/µl blood) for an animal *i* at sampling *j* is explained by the fixed effects *μ* (intercept), *light* (off/on), *concentrate feeding* (yes/no), *plasma cortisol concentration* (ng/ml), *hematocrit* (%), and *sampling* (1–12 in 12 animals or 1–26 in 6 animals) as well as by the random effects *animal identity* (*n*=18), *experimental trial* (*n*=3), and *litter* (*n*=9). The covariance structure for the repeated effect *sampling* was set as first order autoregressive (AR(1)) and for the random effects as scaled identity (ID). The variable *animal identity* designated the subjects in the analysis.
