# Supplementary material for: Data characterizing diurnal rhythms in the number of peripheral CD8α− and CD8α+ γδ T cells in domestic pigs
Source: Data Brief. 2017 Dec 10;16:843–9. doi: 10.1016/j.dib.2017.12.013 (PMC5847622; doi:10.1016/j.dib.2017.12.013)
Supplement: Supplementary file 1 — Supplementary material [file mmc1.pdf]

Declaration of interest

The authors report no conflicts of interest.
